# Novel *Pneumocystis* Antigens for Seroprevalence Studies

**DOI:** 10.3390/jof9060602

**Published:** 2023-05-24

**Authors:** Dora Pungan, Jia Fan, Guixiang Dai, Mst Shamima Khatun, Monika L. Dietrich, Kevin J. Zwezdaryk, James E. Robinson, Samuel J. Landry, Jay K. Kolls

**Affiliations:** 1 John W Deming Department of Internal Medicine, Center for Translational Research in Infection and Inflammation, Tulane University School of Medicine, New Orleans, LA 70112, USA; 2 Department of Biochemistry, Center for Cellular & Molecular Diagnostics, Tulane University School of Medicine, New Orleans, LA 70112, USA; 3 Department of Pediatrics, Tulane University School of Medicine, New Orleans, LA 70112, USA; 4 Department of Microbiology and Immunology, Tulane University School of Medicine, New Orleans, LA 70112, USA; 5 Department of Biochemistry and Molecular Biology, Tulane University School of Medicine, New Orleans, LA 70112, USA

**Keywords:** *Pneumocystis*, pneumonia, serology, glucanase

## Abstract

*Pneumocystis jirovecii* is the most common cause of fungal pneumonia in children under the age of 2 years. However, the inability to culture and propagate this organism has hampered the acquisition of a fungal genome as well as the development of recombinant antigens to conduct seroprevalence studies. In this study, we performed proteomics on *Pneumocystis*-infected mice and used the recent *P. murina* and *P. jirovecii* genomes to prioritize antigens for recombinant protein expression. We focused on a fungal glucanase due to its conservation among fungal species. We found evidence of maternal IgG to this antigen, followed by a nadir in pediatric samples between 1 and 3 months of age, followed by an increase in prevalence over time consistent with the known epidemiology of *Pneumocystis* exposure. Moreover, there was a strong concordance of anti-glucanase responses and IgG against another *Pneumocystis* antigen, PNEG_01454. Taken together, these antigens may be useful tools for *Pneumocystis* seroprevalence and seroconversion studies.

## 1. Introduction

*Pneumocystis jirovecii* pneumonia (PCP) is an opportunistic fungal infection that is severe in immunocompromised patients. The Pneumonia Etiology Research for Child Health (PERCH) study has shown that it is the most common fungal pneumonia in HIV-negative children under the age of five years [1]. In addition, it was among the top ten most common pathogens in cases of pneumonia with a positive chest X-ray and without HIV infection in Mali, Kenya, and Zambia [1]. *P. jirovecii* was more prevalent in children under 1 year old with pneumonia than in those 1 year or older [1]. This high incidence in the first 3–12 months of life coincides with hospitalized PCP pneumonia in children with primary immune deficiencies [2,3]. It has been suggested by other research that *P.jirovecii* DNA can often be found in healthy infants, raising the possibility of humans being a reservoir for the infection within the community [4].

Several seroprevalence studies have used major surface glycoprotein (MSG) antigens to detect PCP because of its strong immune-stimulating properties, its ability to provide protective epitopes, and its involvement in the interactions between *Pneumocystis* and its mammalian host [5]. A limitation of MSG antigens is that they are encoded by several genes and are quite polymorphic [6]. Moreover, the fungus cannot be grown in culture, which restricts the preparations of antigens to organisms of infected host lung, which may be contaminated with other co-infections [5]. Thus, using recombinant antigens facilitated by *P. murina* and *P. jirovecii* genomes may prove to be a more effective tool for seroprevalence studies.

To understand what fungal proteins are produced during *Pneumocystis* pneumonia, we performed proteomics on the BAL fluid of mice with active *Pneumocystis* pneumonia to determine what soluble antigens are released in the lung during active infection. In this analysis, we identified a fungal β-1,3-endoglucanase (PNEG_02407) that has been reported to be expressed in the ascus [7]. Our prior RNAseq data shows expression in both asci and trophs [8]. Interestingly, PNEG_02407 is increased in vivo after antibiotic pressure by the echinocandin class of antifungals [9]. We chose glucanase to explore further due to its high homology among *Pneumocystis* spp. but not other fungi. We expressed recombinant *P. jirovecii* β-1,3-endoglucanase in Chinese hamster ovary (CHO) cells and used this antigen for seroprevalence studies. We found evidence of maternal anti-glucanase in cord blood as well as in the first month of life in two independent pediatric cohorts. This was followed by a nadir in IgG titers in the two pediatric cohorts, followed by an increase over the next two years, which is consistent with known *Pneumocystis* infection in the first 1–2 years of life. Moreover, there was strong concordance with anti-β-1,3-endoglucanase IgG with anti-PNEG_01454 IgG, another antigen we have reported on earlier [10]. Taken together, these data support that *P. jirovecii* β-1,3-endoglucanase is a useful tool for seroprevalence and epidemiology studies of PCP.

## 2. Materials and Methods

### 2.1. Identification of Pneumocystis Antigens in BAL

The BAL samples were from C57BL/6 mice and Rag2-/- mice infected with 100 μL of *P. murina* (~2 × 10^5^ asci) via oral pharyngeal administration, and were collected 2, 4, and 6 weeks post-infection. All animal sites were approved by the Tulane IACUC. Samples were acetone-precipitated with 4X volume, vortexed, and incubated at −20 °C overnight. The solutions were centrifuged to acquire the pellet. Subsequently, the pellet was resuspended and reduced with dithiothreitol. This step was followed by alkylation with iodoacetamide. The mixture was dried (SpeedVac, Eppendorf) and the pellet was subsequently reconstituted in 50 mM Tris-HCl and digested overnight at 37 °C by trypsin at an enzyme to protein mass ratio of 1:50. Peptides were acidified to a final concentration of 0.1% trifluoro-acetic acid (TFA) for SDB-RPS binding, and 20 μg was loaded onto double-stacked SDB-RPS discs with an 18-gauge needle and mounted in a 200 μL tip, as mentioned previously [11]. Briefly, each tip was wetted with 50 μL of 100% acetonitrile and centrifuged at 1000× *g* for 1 min. Following wetting, each StageTip was equilibrated with 50 μL of 30% methanol/1% TFA with centrifugation for each at 1000× *g* for 3 min. Each StageTip was then loaded with the equivalent of ∼20 μg tryptic peptides, followed by washing with 100 µL of 99% propanol and 0.1% TFA for 2 min. The peptides were eluted with 60 µL of elution buffer (80% acetronitrile, 1% ammonium hydroxide), dried, and reconstituted in 2% acetonitrile and 0.1% formic acid. For deep proteome coverage, each sample was fractionated into six fractions by the strong cation ion exchange (SCX). The SCX stage tip was prepared in a similar way to the SDB-RPS tips. The tips were activated with 50 µL acetonitrile and the enriched peptides were loaded, followed by their elution in 6 buffers: SCX buffer 1: 50 mM ammonium acetate, 20% acetonitrile, 0.5% formic acid; SCX buffer 2: 75 mM ammonium acetate, 20% acetonitrile, 0.5% formic acid; SCX buffer 3: 125 mM ammonium acetate, 20% acetonitrile, 0.5% formic acid; SCX buffer 4: 200 mM ammonium acetate, 20% acetonitrile, 0.5% formic acid; SCX buffer 5: 300 mM ammonium acetate, 20% acetonitrile, 0.5% formic acid; SCX buffer 6: 5% ammonium hydroxide, 80% acetonitrile. All the fractions were dried by vacuum centrifugation and suspended in 0.1% formic acid/2% acetonitrile before injection. Each fraction was loaded in LC-MS.

Samples were measured using LC-MS instrumentation consisting of an Ultimate 3000 nanoLC system (Thermo Fisher Scientific, Waltham, MA, USA) coupled, via a nano-electrospray ion source (Thermo Fisher Scientific), to a QExactive HFX Orbitrap (Thermo Fisher Scientific). For DDA, the SCX fractions were loaded onto a 100 lm I.D. × 2.5 cm C18 trap column and PepMapTM RSLC C18 (2 µm, 75 µm × 25 cm) analytical column solvent A (0.1% formic acid in water). The fractions were eluted with a linear gradient from 5% to 30% buffer B over 80 min. Following the linear separation, the system was ramped up to 60% buffer B over 5 min and finally set to 95% buffer B for 8 min, which was followed by re-equilibration to 5% buffer B prior to the subsequent injection. Data were acquired using data-dependent acquisition mode (DDA). After adjusting each fraction to an estimated 0.5–1.0 μg on column, the fractions were measured in a top-12 configuration with 20 s of dynamic exclusion. Precursor spectra were collected from 300–1650 *m*/*z* at 60,000 resolutions (AGC target of 3e6, max IT of 20 ms). The MS/MS were collected on +2H to +5H precursors, achieving a minimum AGC of 2e3. MS/MS scans were collected at 15,000 resolutions (AGC target of 1e5, max IT of 60 ms) with an isolation width of 1.4 *m*/*z* and a NCE of 27.

All LC-MS/MS data were searched using the Sequest HT algorithm within Proteome Discoverer version 2.4.1.15 (Thermo Fisher Scientific) against Mus musculus as the host and *Pneumocystis murina* as the fungal database to obtain peptide and protein identifications. For all searches, trypsin was specified as the enzyme for protein cleavage, allowing up to two missed cleavages. Oxidation (M) and carbamidomethylation (C) were set as dynamic and fixed modifications, respectively. For the Sequest search, the precursor and fragment mass tolerances were set at 10 ppm and 0.5 Da, respectively. Both the peptide spectrum match and protein false discovery rate (FDR) were set to 0.01 and determined using a percolator node.

### 2.2. Production of Glucanase Recombinant Protein

Recombinant *P. jirovecii* β-1,3-endoglucanase was produced in Chinese Hamster Ovarian (CHO) cells by LakePharma/Curia. The recombinant glucanase amino acid sequence is in the supplemental material (Supplemental Figure S1). The formulation buffer of the recombinant protein was 137 mM NaCl, 2.7 mM KCl, 10 mM Na_2_HPO_4_, 2 mM KH_2_PO_4_, pH 7.4. The protein concentration was 1.61 (mg/mL) and the physical state was frozen and stored at −80 °C.

### 2.3. Antigen Serology ELISA

For the seroprevalence study, we tested residual blood samples that were collected between 18 March and 15 May 2020, from children ≤ 18 years of age from Children’s Hospital in New Orleans, Louisiana. Collection and processing have previously been described [12]. All serum samples were SARS-CoV-2 -negative and HIV-negative [12]. For the ELISA assays, Maxisorp plates (ThermoFisher cat. no. 442404) were coated with 2 µg of *Pneumocystis* antigen in 100 µL of 1X sterile phosphate buffered saline (PBS) per well overnight at 4 °C. Plates were aspirated and washed twice. Plates were blocked with 200 µL of 2X PBS with 1% Tween 20 and 10% BSA (Invitrogen, 88-50470, Waltham, MA, USA) for two hours at room temperature. Plates were aspirated and washed twice. Plates were then stained with 50 µL sample serum diluted 1:32 in 1X PBS with 1% Tween-20 and 10% BSA, covered, and incubated for 2 h at room temperature on a microplate shaker set at 400 rpm. Plates were aspirated and washed four times. For measuring IgG, goat anti-human IgG HRP (SouthernBiotech, cat. no. 2040-05, Birmingham, AL, USA) was diluted 1:4000 in 1X PBS with 1% Tween-20 and 10% BSA, and 100 µL was added to all wells. For measuring IgM, goat anti-human IgM HRP (SouthernBiotech, cat. no. 2020-05) was diluted 1:4000 in 1X PBS with 1% Tween-20 and 10% BSA, and 100 µL was added to all wells. Plates were covered and incubated for 1 h at room temperature on a microplate shaker set at 400 rpm. Plates were aspirated and washed four times. A 100 µL volume of tetramethylbenzidine (TMB) substrate solution (Invitrogen, 88-50470) was added to each well and incubated at room temperature for 1–5 min, depending on the control serum. The reaction was stopped with 50 µL of 2N H_2_SO_4_ added to each well. The optical density at 450 nm (OD450) and 570 nm (OD570) was read using a BioTek Synergy HT microplate reader. The OD reported is the reading at 450 nm minus the reading at 570 nm to remove background.

For human cord blood donors (9), we screened cord blood donors (StemCell Technologies, Vancouver, BC, Canada) using the same ELISA method as previously described, except the serum was serially diluted 1:10–1:320 and the detection antibody used was goat anti-human IgG HRP (SouthernBiotech, cat. no. 2040-05) diluted 1:4000 in 1X PBS with 1% Tween-20 and 10% BSA, and 100 µL was added to all wells.

To assay convalescent titers, we infected C57BL/6 mice with 100 μL of *P. murina* inoculum (~2 × 10^5^ asci) via oral pharyngeal administration. Two or 6 weeks later, we collected the sera and tested it for IgG anti-glucanase using the same ELISA method as described above, except the sera were tested at a 1:200 dilution, and the detection antibody was goat anti-mouse IgG HRP (SouthernBiotech, cat. no. 1030-05) diluted 1:4000 in 1X PBS with 1% Tween-20 and 10% BSA.

### 2.4. Statistical Analysis

**Figure 1:**
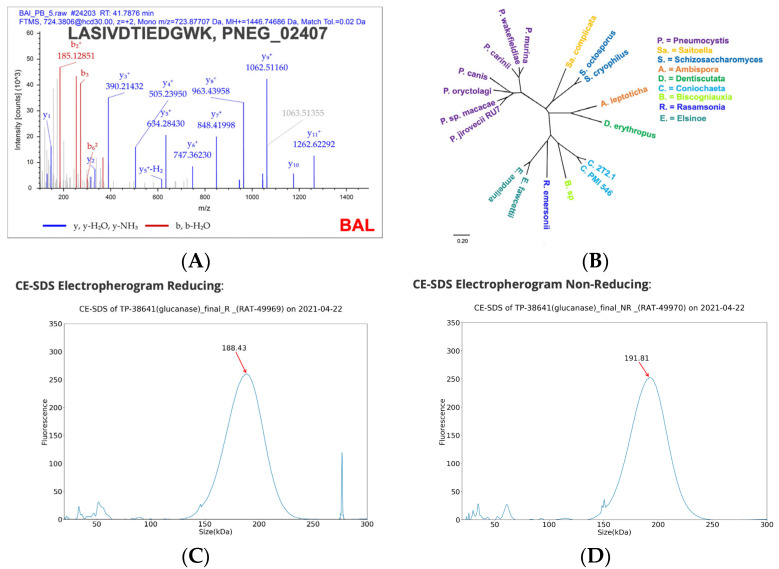
Identification of glucanase and recombinant glucanase production. (**A**) Sequence confirmation of *P. murina* protein. LC-MS/MS data were searched using the Sequest HT algorithm within Proteome Discoverer 2.4 against Mus musculus as the host and Uniprot *Pneumocystis* murine stain B123 reference database (UP000011958) as the fungal database to obtain peptide and protein identifications. (**B**) Phylogenetic analysis of glucanase protein, demonstrating conservation in *Pneumocystis* spp. (**C**) CE-SDS electropherogram of recombinant *P. jirovecii* glucanase protein under reducing conditions. (**D**) CE-SDS electropherogram of recombinant *P. jirovecii* glucanase protein under non-reducing conditions.

The phylogenetic tree (Figure 1B) was built with the use of Blast Search in MEGA11 following the pipeline described [13], and inputting the *P. jirovecii* glucanase protein sequence (GeneBank Accession: XP_018229679., Supplemental Figure S1). Statistical analyses were conducted utilizing GraphPad Prism (version 9.5.1). The difference in antibody titers in the pediatric cohort was assessed by comparing the 1:32 OD values via the Mann–Whitney test. Any *p* values that were less than 0.05 were viewed as significant.

## 3. Results

### 3.1. Identification of Pneumocystis Antigens in BAL

As outlined in the Methods, we performed proteomics in the BAL fluid of infected B6 mice and identified several *Pneumocystis* proteins in the BAL, including several major surface glycoproteins as well as PNEG_02407, a putative endo-beta-1,3-glucanase (MS depicted in Figure 1A) [13]. We prioritized this protein, as it is highly conserved in *P. jirovecii* and other *Pneumocystis* spp. but not in environmental fungi (Figure 1B). In addition, it has been reported to be more highly expressed in trophic forms [9], which are the forms that attach to type I pneumocytes [14]. We were able to successfully express and purify recombinant glucanase expressed in CHO cells, as depicted in Figure 1C,D.

### 3.2. Antigen Serology in Pneumocystis-Infected Mice

**Figure 2:**
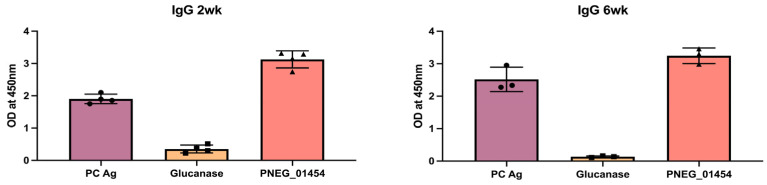
Convalescent antibody responses after *P. murina* infection. Anti-whole *Pneumocystis* antigen (PC Ag), Anti-glucanase, and anti-PNEG_01454 IgG detected after *P. murina* infection in a murine model. C57BL/6 mice were infected with 100 μL of *P. murina* inoculum (~2 × 10^5^ asci) via oral pharyngeal administration. 2 or 6 weeks later, sera were collected and measured IgG by ELISA. Serum dilution: 1:200.

IgG responses to *Pneumocystis*-specific antigens were assayed by ELISA two weeks and six weeks after C57BL/6 mice were infected with *P. murina* (Figure 2). PC-infected mice generated significant anti-PNEG_01454 and anti-whole *Pneumocystis* antigen (PC antigen) IgG, as well as lower titers of anti-glucanase IgG. PC antigen is a whole antigen preparation and thus has more reactivity. PNEG_01454, interestingly, is recognized by germ line IgM, and the higher titer may reflect a larger precursor pool of antigen-specific B cells prior to infection [10].

### 3.3. Antigen Serology in Pediatric Cohorts

**Figure 3:**
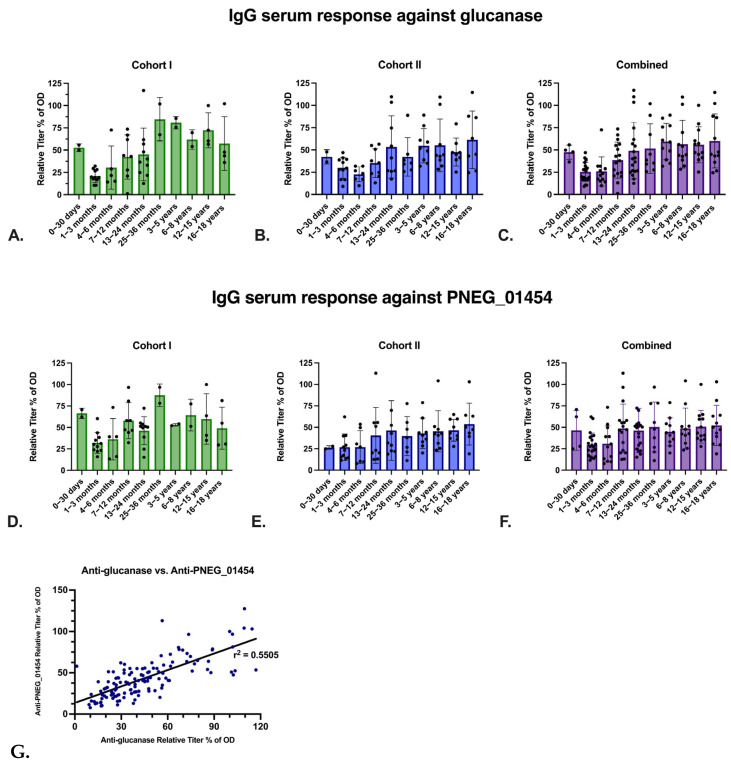
Anti-*P. jiorvecii* glucanase and anti-PNEG_01454 IgG detected in pediatric human samples measured by ELISA. Serum dilution: 1:32. (**A**) Cohort 1 anti-glucanase IgG. (**B**) Cohort 2 anti-glucanase IgG. (**C**) Cohorts 1 and 2 combined anti-glucanase IgG. (**D**) Cohort 1 anti-PNEG_01454 IgG. (**E**) Cohort 2 anti-PNEG_01454 IgG. (**F**) Cohorts 1 and 2 combined anti-PNEG_01454 IgG. (**G**) Simple linear regression of IgG anti-glucanase titer vs. IgG anti-PNEG_01454 titer of pediatric human samples. *p*-value: <0.0001.

A total of 133 pediatric blood samples, consisting of a discovery and validation cohort, were obtained from Children’s Hospital in New Orleans from 18 March to 15 May of 2020. Subjects were HIV-negative and SARS-CoV-2-negative. We detected the presence of anti-glucanase IgG responses by ELISA in Cohort 1 (Figure 3A), which showed the presence of anti-glucanase IgG responses in the first month of life (consistent with maternal IgG), followed by a nadir at 1–3 months of age. The validation cohort (Cohort 2, Figure 3B,C) from the same pediatric center similarly showed neonatal IgG levels declined, reaching a nadir at 4–6 months of age. Given the fact that there was anti-glucanase IgG at birth, we verified that there was anti-glucanase IgG detected in an independent sample of human cord blood donors (9), and all donors tested were positive (Supplemental Figure S2).

We compared the glucanase IgG responses in the 1–3 month of age samples from both cohorts with the values from 0–30 days of age responses using the Mann–Whitney U test; *p* value is 0.0007. The nadir was then followed by an increase over time, and 114 of the 133 samples (85.71%) were positive (OD > 0.2; 20.58% relative titer of OD). To determine if this was potentially due to *Pneumocystis* infection, we also assayed IgG responses to the putative cell adhesion protein PNEG_01454 (Figure 3D–F) and we observed a high concordance of IgG. We also measured anti-glucanase IgM and, consistent with the known polyreactivity of IgM, we observed low titers in the first year of life, with increases in older subjects. In contrast to IgG, we did not observe a nadir.

### 3.4. Possible CD4+ T-Cell Epitopes of P. jirovecii β-1,3-Endoglucanase

Possible CD4+ T-cell epitopes of *P. jirovecii* β-1,3-endoglucanase were identified on the basis of antigen processing likelihood (APL) combined with MHCII-peptide binding affinity. The APL algorithm uses a protein’s 3D structure and amino-acid sequence conservation to generate profiles of conformational stability and then evaluates the likelihood that a particular peptide will be processed from the native protein and presented by an MHCII protein to CD4+ T cells [15]. In general, peptides within conformationally stable segments adjacent to flexible, protease-sensitive segments obtain the highest APL scores. Residue-specific APL values were averaged for a series of 15-residue peptides spanning the length of *P. jirovecii* glucanase in 5-residue steps. For each peptide, MHCII-peptide binding affinity [1-log50k(aff)] was predicted for several common class II HLA molecules (DPA1*01:03-DPB1*04:01, DRB1*15:01, DRB1*04:01, and DRB1*01:01) using the NETMHCII (2.3) server [16], or the median binding-affinity rank was predicted for a 7-allele HLA reference set using the IEDB server [17,18]. Ultimately, the epitope probability of each peptide was calculated as the weighted combination of z-scores representing APL (31%) and MHCII binding (69%) for each HLA molecule or the HLA reference set. 

Since no 3D structure has been reported for *P. jirovecii* β-1,3-endoglucanase, a homology model was created using automated functions of the SWISS-MODEL server [19]. SWISS-MODEL identified a single X-ray crystal structure (PDB:4K35) that could serve as a template, a β-1,3-glucanase from the fungus R. miehi. Alignment of the template and target glucanases revealed well-distributed sequence identity (34% overall) spanning residues 89–785 of the 788-residue *P. jirovecii* glucanase. An AlphaFold of *P. jirovecii* β-1,3-endoglucanase was also available, but it was not selected for analysis by APL because it included segments that had poor confidence and were disordered in the R. miehi structure. We note that the well-ordered portions of the AlphaFold and the SWISS-MODEL were virtually identical, and backbones could be aligned with an average RMSD of 1.2 angstroms.

**Figure 4:**
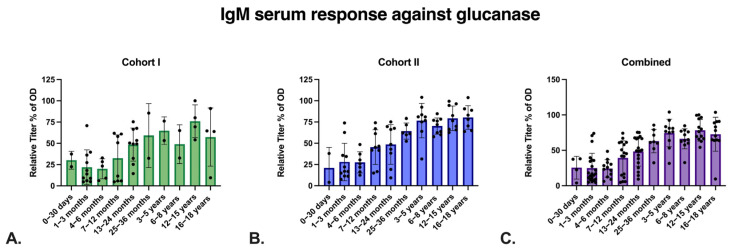
Anti-*P. jirovecii* glucanase IgM detected in pediatric human samples measured by ELISA. Serum dilution: 1:32. (**A**) Cohort 1 anti-glucanase IgM. (**B**) Cohort 2 anti-glucanase IgM. (**C**) Cohorts 1 and 2 combined anti-glucanase IgM.

**Figure 5:**
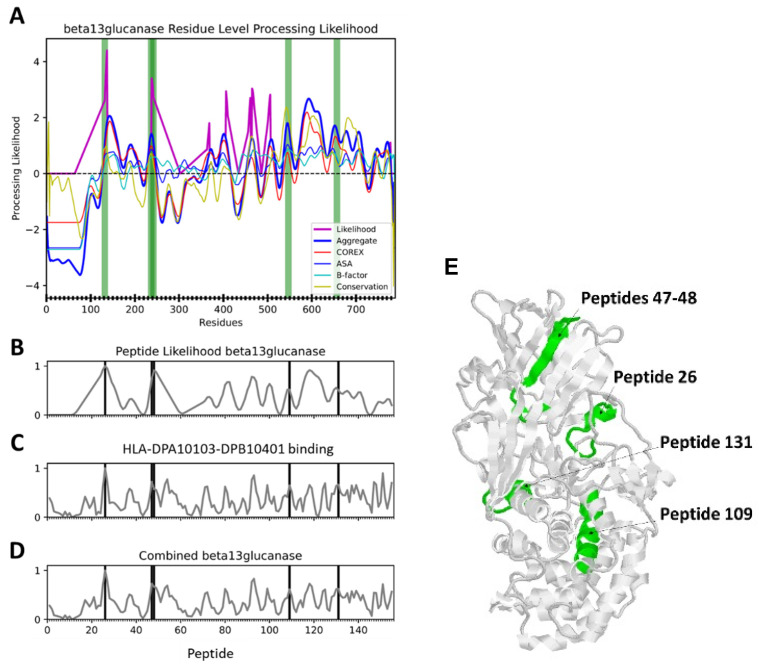
Example of epitope probability in *P. jirovecii* β-1,3-glucanase with presentation by HLA-DPA1*01:03-DPB1*04:01. (**A**) Antigen processing likelihood (APL) and conformational stability by residue. (**B**) APL by peptide. (**C**) Predicted affinity of MHCII binding. (**D**) Combined APL and MHCII binding. (**E**) Locations of predicted CD4+ epitopes in the structure of *P. jirovecii* β-1,3-glucanase.

Five highly probable CD4+ epitopes that are expected to exhibit promiscuous dominance were identified as having a combined APL/MHCII-binding score within the 80th percentile for each of the tested HLA-binding systems (Figure 4 and Figure 5 and Table 1). Additionally, when APL and MHCII binding were assessed independently, these five peptides also scored in the 70th percentile of APL and the 80th percentile of each HLA system. For using the peptides for diagnostics, this would only work if the peptides are sufficiently different from corresponding sequences in commensal or other pathogen proteins. Peptides 26 and 109 are conserved and thus would not be suitable, but the other two segments, 47–48 and 131, should be suitable (Table 1).

## 4. Discussion

Recent prospective studies to assess pneumonia etiology in children suggest that *Pneumocystis* is a frequent infection in HIV-negative children under 2 years of age. These data are consistent with prior autopsy studies documenting the high prevalence of *Pneumocystis* infection in the lungs of infants [4,5,19], and the level of *Pneumocystis* was also associated with type 2 immune responses in the lung, as assessed by the induction of mucin genes [20]. This type 2 response is also observed in the murine model [21,22].

The PERCH study used PCR on throat swabs to classify *Pneumocystis* pneumonia, but currently there are no robust tools to document seroprevalence and seroconversion studies. Prior serological studies used non-recombinant antigens, and due to the inability to grow *Pneumocystis* ex vivo, these preparations may be contaminated with host-derived proteins [5]. Our findings add to this prior study, which also reported sequential changes in levels of antibody against MsgC and MsgA during early childhood and that higher antibody responses occurred in children who experienced more respiratory infections [5]. Thus, the identification of proteins/recombinant antigens may provide the field with more accurate tools to assay seroprevalence. 

To this end, we conducted unbiased proteomics in the BAL fluid of infected mice and identified a conserved fungal glucanase. This protein expressed well in CHO cells and was recognized by both human cord blood as well as in pediatric samples. The level of anti-glucanase IgG is consistent with PERCH and other studies that have assessed *Pneumocystis* prevalence in pediatric populations. Moreover, there was strong concordance with anti-glucanase IgG and IgGs against PNEG_01454 [10]. Thus, we believe that glucanase may represent an important antigen on which to conduct seroprevalence studies. 

## 5. Patents

Tulane University has filed a provisional patent application related to this work. 

## Figures and Tables

**Table 1:** Putative CD4+ T-cell epitopes in *P. jirovecii* glucanase.

Peptide No.	Peptide Sequence	Over 80% Identical	Start	End
26	HTNKFYANFFLGTQE	200 hits	126	140
47	VRGAAYITAIYSDLT	Macacace and wakefieldiae	231	245
48	YITAIYSDLTPLFTS	Macacace and wakefieldiae	236	250
109	HHFHYGYMIFAAAII	200 hits	541	555
131	RSLHSYFLYESSNTI	24 hits	651	665

## Data Availability

If accepted for publication, the data in this paper will be made publicly available per NCI/U54 policy.

## Supplementary Materials

The following supporting information can be downloaded at: https://www.mdpi.com/article/10.3390/jof9060602/s1, Figure S1: Recombinant *P. jirovecii* glucanase sequence; Figure S2: Anti-glucanase IgG detected in cord blood samples measured by ELISA.
