# Supplementary material for: Novel Pneumocystis Antigens for Seroprevalence Studies
Source: J Fungi (Basel). 2023 May 24;9(6):602. doi: 10.3390/jof9060602 (PMC10300987; doi:10.3390/jof9060602)
Supplement: Supplementary file 1 [file jof-09-00602-s001.zip › jof-2311125-supplementary.pdf]

**Supplemental Material:**

**Supplemental Figure S1: Recombinant *P. jirovecii* glucanase sequence:**

METDTLLLWVLLLWVPGSTGVNNTLDHDRSITLRTNSTNTINNTNTSSTEKTLTPTKTVPDKRSTI
SPDIYKPINVFQPITERPPLSIFENVTHPLQPKSIFDGDLTKPIHTNKFYANFFLGTQEFPTYLDPY
VVTWVPGNYSGITVSHVDDYQKVFASDNPPSYFLSPLGIYSVVFSAQELNNGILALSSLDQMSV
NVTITPKNSTEKKMSLPLVRGAAYITAIYSDLTPLFTSVVGFKKIEKVEIKNYNYFKFIITLYDEKRW
LLYAFPEKKTLFNLEIQNNILKATCGAFNGTIQVTKVPINNSDAENILDASAGTYAKKITLSAIVDGK
TGNYTYTFDIFQYKGHSLLHYAMPHHMSSFDNITASKRTNVSLPSTTNGLMVAYVGECWNMIEH
DLPVDIDFFPYSQGKEPEYSDEALKAIKEAAMYELSQDFDSQIDKNTIYFSGKVFSKFALLCLVIN
NILKNKTLAEECLKKFNHSYMPFVENLNEYKLVYDTTWNGIVTDQGFTKGPLADFGASYYNDHH
FHYGYMIFAAAIISYIDPAWAEKVKDWILDLIRDVANPVHDSYFPAFRSFDWFTGHSWAKGIFESP
DGKDEESSSEDYNFYFAMKLFGMTIGDNAMVSRANLILAILKRSLHSYFLYESSNTIMPRVFLPN
YVSGIKFMNKIHHTTYFSPRLECIQGIHMLPLTAISAYIRTPSFVQSEWDNKLASIINTINDGWKGIL
FANLAISKPKESYDFFSSCFDRKYLDNGSSLAWYLVYSSAFANSAESGGGGAGGGGHHHHHH
HHHH

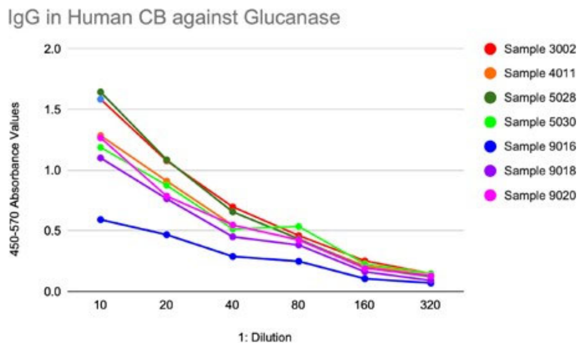


**Supplemental Figure S2.** Anti-glucanase IgG detected in Cord Blood Samples measured by ELISA. Serum serially diluted 1:10-1:320
